# Telmisartan prevents high-fat diet-induced hypertension and decreases perirenal fat in rats

**DOI:** 10.7555/JBR.26.20120013

**Published:** 2012-05-18

**Authors:** Yaping Wang, Yan Song, Meng Suo, Xin Jin, Gang Tian

**Affiliations:** Department of Cardiology, the First Affiliated Hospital of Medical College, Xi'an Jiaotong University, Xi'an, Shaanxi 710061, China.

**Keywords:** telmisartan, high-fat diet, hypertension, perirenal fat, adiponectin

## Abstract

We sought to investigate the effects of telmisartan on high-fat diet-induced hypertension and to explore the possible underlying mechanisms. Rats receiving high-fat diet were randomly divided into two groups, the telmisartan group (*n* = 9) and the high-fat diet group (*n* = 10). The control group consisted of age-matched rats on a regular diet (*n* = 10). At the end of the treatment, the body weight, blood pressure, insulin sensitivity and serum adiponectin levels of all rats were examined, and their visceral fat was extracted and weighed. Our results showed that telmisartan improved insulin resistance and dyslipidemia and increased serum adiponectin levels. Telmisartan also lowered both systolic blood pressure and diastolic blood pressure, and decreased the accumulation of perirenal fat associated with high-fat diet. Furthermore, telmisartan increased *adiponectin* mRNA expression in the perirenal fat. Correlation analysis showed that both systolic blood pressure and diastolic blood pressure were positively correlated with perirenal fat. These effects of telmisartan may be mediated through decreases in perirenal fat and contributed to the improvement of perirenal fat function. Our findings suggested a strong link between perirenal fat and high-fat diet-induced hypertension, and identified telmisartan as a potential drug for the treatment of obesity-related hypertension.

## INTRODUCTION

The increasing prevalence of overweight and obesity is recognized as a serious public health problem worldwide[1],[2]. In 2008, a systematic analysis of epidemiological studies spanning 199 countries revealed that an estimated 1.46 billion adults were overweight, of whom 502 million were obese[1]. According to the data from the Framingham Cohort, obesity possibly accounted for 78% and 65% of essential hypertension in men and women, respectively[3]. Most studies suggested that visceral body fat is an important determinant of blood pressure elevation in both men and women[4]-[6]. Obesity, especially abdominal obesity, is strongly associated with hypertension[7]. The high prevalence of obesity and other related disorders such as hypertension, insulin resistance, type 2 diabetes, hyperlipidemia and subsequent cardiovascular diseases are great challenges for global medical and health network. On the other hand, increasing rate of weight gain in the population is an indicator of increased burden on the society in the long run[8],[9].

Hypertension due to obesity is primarily related to visceral obesity, which is associated with adipose dysfunction, insulin resistance and dyslipidemia. Effective approaches for treating obesity-related hypertension should include not only hypertension, but also metabolic disorders associated with obesity. For instance, several studies have shown that telmisartan, an angiotensin receptor blocker (ARB) and partial agonist of peroxisome proliferator-activated receptorgamma (PPAR-γ), can reduce blood pressure as well as improve insulin resistance in obese subjects[10]-[12]. However, the mechanisms by which it acts are still unknown.

Visceral fat is a complex metabolic and endocrine organ, and accumulation of visceral fat plays an important role in the pathogenesis of hypertension, insulin resistance, and abnormal secretion of adipokines[13]-[15]. Recent studies have found that different adipose deposits have different roles[16] and relationships to the occurrence and development of high-fat induced hypertension[17]. Perirenal fat accumulates on the kidneys and tightly encapsulates them in obese individuals, which may damage renal function[18],[19]. Therefore, we hypothesized that perirenal fat plays an important role in the development of high-fat diet-induced hypertension. To test this hypothesis, we established a high-fat diet-induced animal model to investigate the effects of telmisartan on blood pressure, body weight, metabolic parameters, serum adiponectin, and changes in perirenal fat weight and secretion. Additionally, we analyzed the correlation among perirenal fat, blood pressure and epididymis fat.

## MATERIALS AND METHODS

### Animals and experimental design

Thirty mature male Wistar rats (Beijing Vital River Laboratories Animal Technology Co., Beijing, China) of SPF grade, weighing from 200 to 220 g and aged 8 weeks, were housed in a light, temperature, and humidity controlled room (12-h light, 12-h dark cycle, lights on/off at 07:00 and 19:00, respectively; 21±1°C, and 55%±5% relative humidity). After one week of acclimation, the rats were allowed *ad libitum* access to 56% high-fat food (7.0% protein, 37% carbohydrate, and 56% fat, 21.8 kJ/g, *n* = 20) and water. Ten age- and body weight-matched rats with standard laboratory food (23.3% protein, 66.5% carbohydrate, 10.2% fat, 13.4 kJ/g, *n* = 10) were used as controls (CON). The rats went through a 12-week dietary intervention. The experimental protocols were approved by the local institutional review board at the authors' affiliated institutions and animal studies were carried out in accordance with the established institutional guidelines regarding animal care and use.

Telmisartan (DongRui, Suzhou, China) was dissolved in phosphate-buffered saline (PBS) containing 5 g/L carboxymethyl cellulose. Each solution was prepared on the day it was administered. Model rats were randomly divided into two groups including the telmisartan group (high-fat telmisartan, HFT, *n* = 10) and the non-treatment group (High-fat, HF, *n* = 10). Rats of the HFT group and the HF group continued to receive high-fat diet until the end of the study. The HFT group was administered with telmisartan by gastric lavage at 8 mg/kg per d for 20 consecutive weeks. The rats of the HF and CON group received untreated PBS containing 5 g/L carboxymethyl cellulose. One rat receiving telmisartan died from gavage during the course of the experiment.

### Physiological and biochemical measurements

The rats were weighed after fasting for 14 h every week, and every weight measurement was performed twice to calculate an average weight. After 20 weeks of treatment, blood was withdrawn from the caudal vein after fasting for 14 h. Blood samples were collected and sera were removed from centrifuged samples and snap frozen at -20°C until assayed. Levels of total cholesterol (TC), triglycerides (TG), high-density lipoprotein cholesterol (HDL-C) and low-density lipoprotein cholesterol (LDL-C) were determined using an automatic biochemical analyzer. Plasma glucose was measured in duplicate by glucose oxidase method. Insulin levels were measured using the radioimmunoassay (RIA) method. The homeostasis model assessment index of insulin resistance (HOMA-IR) was calculated as described in previous studies[20],[21] by using the formula: fasting plasma glucose (FPG, mmol/L)×fasting insulin (FINS, mIU/L)/22.5. Serum adiponectin levels were determined by enzyme-linked immunosorbent assay (ELISA) following the manufacturer's instructions (Dongxiong, Shanghai, China). As rats were obese and had thick tails, it was difficult to use the tail-cuff method. Blood pressure was measured via carotid artery intubation at the end of treatment. Carotid pressure was measured continuously in anesthetized rats by using a BL-420F biological signal collecting and processing system (Taimeng Biotech. Co., Chengdu, China), and blood pressure was calculated using the software provided by the manufacturer. The rats were sacrificed to dissect their perirenal and epididymis fat. The fat samples were cleaned of blood in icing physiological saline, blotted and weighed immediately, and then frozen in liquid nitrogen and stored at -80°C until mRNA extraction.

### Real-time quantitative PCR

Adiponectin was amplified by PCR and quantified using real-time quantitative PCR as follows. Total RNA was prepared from perirenal fat tissues using TRIzol (Invitrogen, Carlsbad, CA, USA). The quantity and quality of isolated RNA was determined. Complementary deoxyribonucleic acid (cDNA) was synthesized from 150 ng of total RNA using a reverse transcriptase kit (Takara Shuzo Co. Ltd., Shiga, Japan) following the manufacturer's instruction. Expression of *β-actin* mRNA was used as the control. Primer sequences, which are listed below, were designed and provided by Takara: *adiponectin*, 5′-GGAAACTTGTGCAGGTTGGATG-3′ (sense), and 5′-GGGTCACCCTTAGGACCAAGAA-3′ (antisense), *β-actin*, 5′-ATCTGGCACCACACACCTTC-3′ (sense), 5′-ATCTGGCACCACACACCTTC-3′ (antisense). Real-time PCR was performed using SYBR Premix Ex *Taq*™ (Takara) following the manufacturer's instruction. The copy number for each transcript was normalized to β-actin expression. Fluorescence spectra were recorded during the elongation phase of each PCR cycle. Expression of mRNA was calculated by the 2^−ΔΔCt^ metho d. To confirm amplification specificity, every sample was performed in triplicate.

### Statistical analysis

All analyses were performed using the SPSS for Windows (Version 13.0; SPSS Inc., Chicago, IL, USA). Data were expressed as mean±SME. for quantitative values. Unless otherwise noted, data were analyzed using analysis of variance (ANOVA) followed by Tukey's *post hoc* tests. Correlation analysis was performed by calculating Spearman's rank correlation coefficient. *P* < 0.05 was considered statistically significant.

## RESULTS

### Effects of telmisartan treatment on body weight, blood lipid spectrum, FPG and insulin resistance

By the end of this study, as we expected, body weight gain, FPG and calculated HOMA-IR in the HF group were significantly higher than those of the control group (all *P* < 0.05). Under high-fat diet, long-term (20 weeks) treatment with telmisartan decreased the body weights of rats, which were significantly lower than those of the HF and control group (*P* < 0.05 in both, ***Table 1***). Furthermore, telmisartan treatment decreased the levels of FPG and HOMA-IR, and improved insulin resistance compared with the HF group. The blood lipid spectrum of high-fat diet animals was significantly different from that of the control group, while TC, TG and LDL-c increased, HDL-c decreased in high-fat diet animals (*P* < 0.05, ***Table 1***). Telmisartan treatment improved abnormality of plasma lipid and decreased the level of TG and LDL, but showed little effect on the level of TC and HDL-C.

**Table 1:** Effects of telmisartan on body weight, FPG and HOMA-IR, and levels of blood lipid components

Group	Body weight (g)	FPG(mmol/L)	HOMA-IR	TC(mmol/L)	TG(mmol/L)	LDL-C	HDL-C(mmol/L)
Control (*n* = 10)	713.10 ± 8.85	6.02 ± 1.08	7.12 ± 1.38	6.12 ± 1.16	0.85 ± 0.32	1.23 ± 0.43	2.12 ± 0.24
HF (*n* = 10)	791.20 ± 10.39*	9.37 ± 1.03*	24.03 ± 2.67*	7.67 ± 0.41*	1.31 ± 0.59*	2.11 ± 0.80	1.52 ± 0.33*
HFT (*n* = 9)	694.00 ± 10.56^#^	7.49 ± 1.29^#^	9.81 ± 1.84^#^	7.60 ± 0.58*	0.99 ± 0.58^#^	1.16 ± 0.34^#^	1.52 ± 0.36*

Note: mice in the HF group received high fat diet and in the HFT group received high fat diet and telmisartan. *P < 0.05, *vs* the control group; ^#^*P* < 0.05, *vs* the HF group. FPG: fasting plasma glucose; HOMA-IR: homeostasis model assessment index of insulin resistance; HDL-C: high-density lipoprotein cholesterol; LDL-C: low-density lipoprotein cholesterol; TC: total cholesterol; TG: triglyceride.

### Effects of telmisartan on blood pressure

**Fig. 1:**
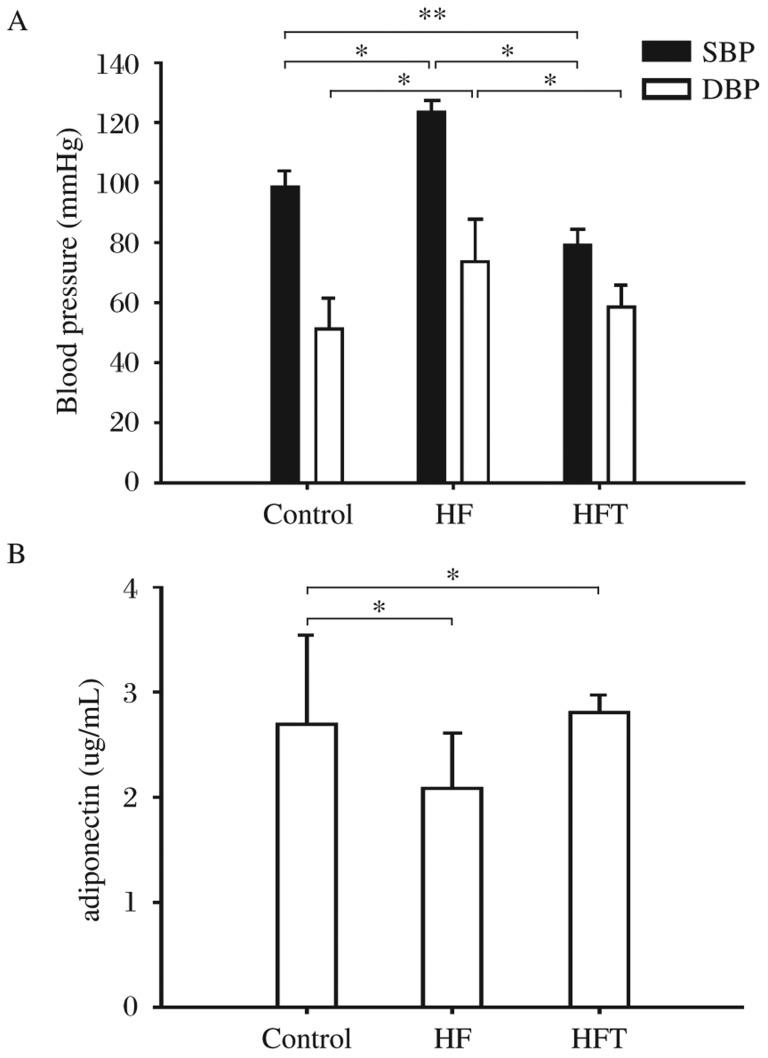
Effects of telmisartan treatment on blood pressure (A) and serum level of adiponectin (B). The rats were treated with normal diet (the control group, *n* = 10), high-fat diet only (the HF group, *n* = 10) and high-fat diet with telmisartan (the HFT group, *n* = 9) *P < 0.05. SBP: systolic blood pressure; DBP: diastolic blood pressure.

Both SBP and DBP were significantly higher in the HF group compared with the control group. As expected, telmisartan decreased both SBP and DBP in the HFT group compared to the HF group. DBP in the HFT group was comparable to the control group while SBP was lower than that of the control group (*P* < 0.05, ***Fig. 1***).

### Effects of telmisartan on serum adiponectin

As reported in previous studies[22],[23], high-fat diet decreased serum adiponectin (*P* < 0.05) in the HF group. In the HFT group, after 20 weeks of telmisartan treatment, the level of serum adiponectin was increased and comparable to the control group (***Fig. 1B***).

### Effects of telmisartan on weights and *adiponectin* mRNA of perirenal and epididymis fat

**Fig. 2:**
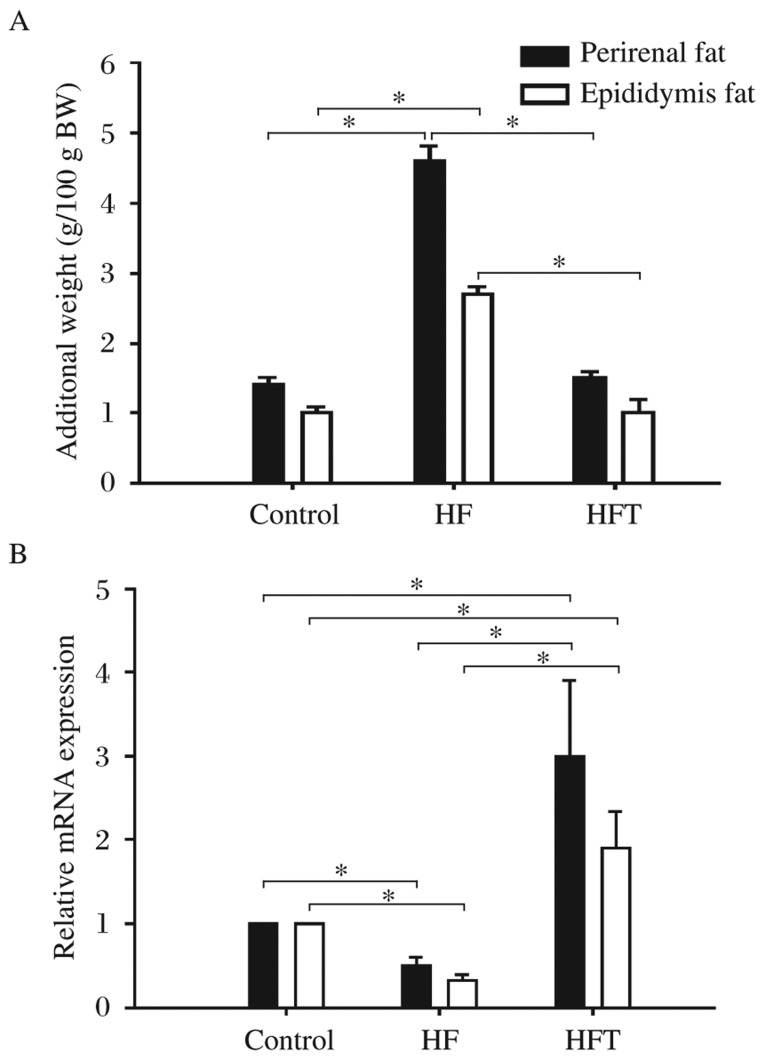
Effects of telmisartan on weights and *adiponectin* mRNA of perirenal and epididymis fat. The rats were treated with normal diet (the control group, *n* = 10), high-fat diet only (the HF group, *n* = 10) and high-fat diet with telmisartan (the HFT group, *n* = 9). *P < 0.05.

As shown in ***Table 1***, significant differences were found in the body weights of animals in the three groups. In order to exclude the possible effects of body weight, the weights of perirenal and epididymis fat were adjusted for the body weight of each animal. The results showed that the amount of both perirenal and epididymis fat increased in the HF group compared to that of the control group (*P* < 0.05), and telmisartan treatment reversed the increase in the amount of perirenal (*P* < 0.01) and epididymis fat (*P* < 0.05) caused by high-fat diet (***Fig. 2A***).

The results of our RT-PCR assays revealed that the mRNA levels of adiponectin decreased in both perirenal fat and epididymis fat in mice receiving high-fat diet (*P* < 0.05). Interestingly, telmisartan treatment increased adiponectin mRNA expression significantly in both perirenal and epididymis fat (*P* < 0.01 in both) (***Fig. 2B***), but the increase of *adiponectin* mRNA expression in perirenal fat was more significant.

### Correlation analysis of blood pressure and body weight, and additional weights of perirenal fat and epididymis fat

**Fig. 3:**
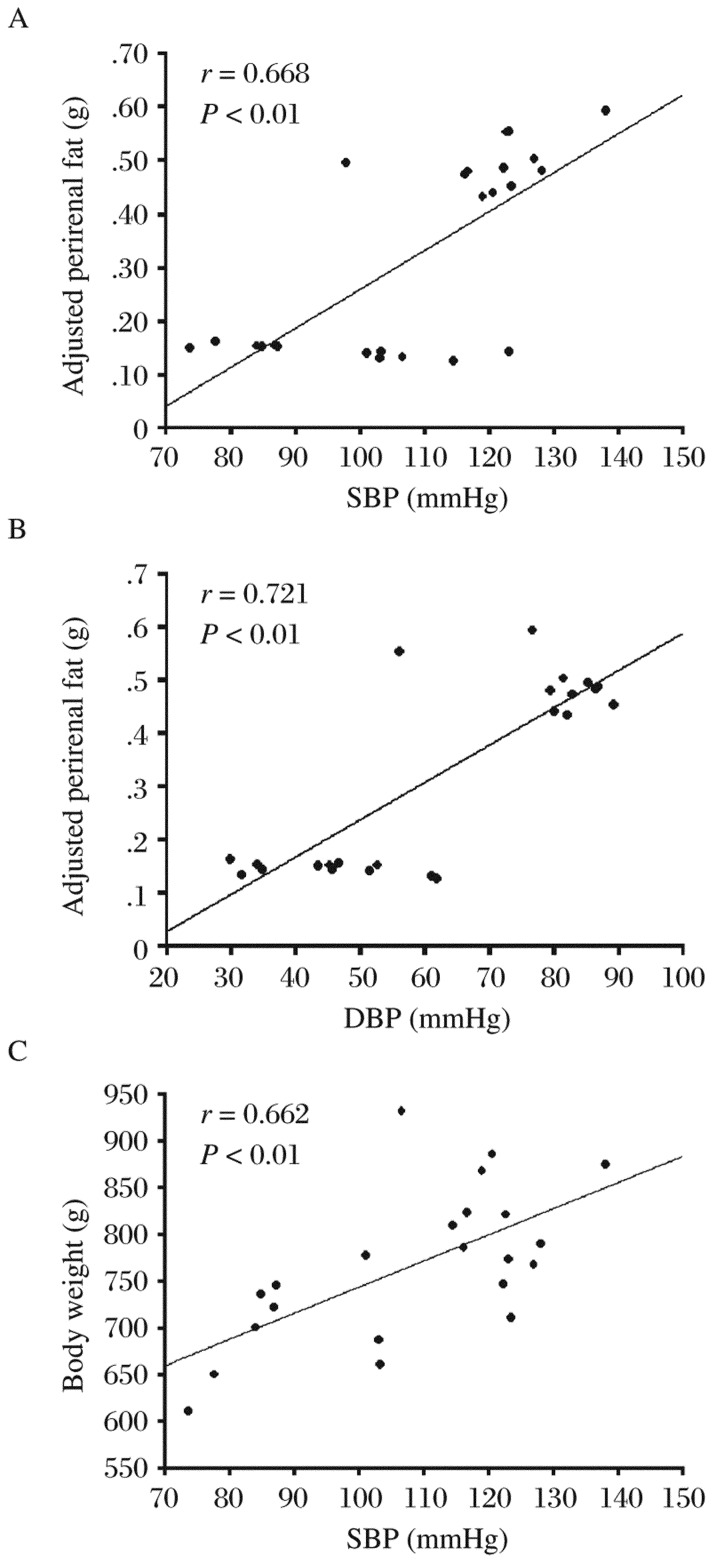
The linear correlation and scatter diagrams of blood pressure and body weight, and blood pressure and weight of visceral fat. Scatter diagram of systolic blood pressure (SBP) and additional perirenal fat are shown in A. Scatter diagram of diastolic blood pressure (DBP) and additional perirenal fat are shown in B. Scatter diagram of SBP and body weight are shown in C.

We analyzed the linear correlation of blood pressure with the whole body weight as well as the additional weights of perirenal and epididymis fat. Our results showed that both SBP and DBP were positively correlated with the adjusted weights of perirenal fat (both *P* < 0.01) (***Table 2***, ***Fig. 3A*** and ***3B***). We also found that SBP, but not DBP, was correlated with the total body weight (*P* < 0.01) (***Table 2***, ***Fig. 3C***). Furthermore, no correlation was found between the adjusted epididymis fat weight and blood pressure.

**Table 2:** Correlation of blood pressure with total body weight and additional weight of visceral fat

Variables	Body weight	Additional weight of perirenal fat	Additional weight of epididymis fat
SBP	0.662^a^	0.668^a^	0.306
DBP	0.253	0.721^a^	0.276

Data indicated by Spearman's rank correlation coefficient. The additional weights of perirenal and epididymis fat were calculated by adjusting for the body weight of each animal. ^a^*P* < 0.01. DBP: diastolic blood pressure; SBP: systolic blood pressure.

## DISCUSSION

In the present study, we found that rats on a high-fat diet have significant disturbances in glucose and lipid metabolism as reported in previous studies[22],[24]. Telmisartan administration improved insulin resistance, treated metabolic disorders, and increased serum levels of adiponectin. In addition to being a blocker of angiotensin II receptor 1, telmisartan is also a partial agonist of PPAR-γ. Telmisartan treatment *in vitro* has been shown to increase the expression of PPAR-γ as well as target genes, including adipocyte fatty acid-binding protein (aP2), adiponectin, and acetyl coenzyme A (CoA) carboxylase in obese murine models and humans[23],[25],[26]. Since PPAR-γ is a target for insulin-sensitizing agents, activation of PPAR-γ induced by telmisartan would be expected to improve insulin resistance and hyperglycemia in animals with high-fat diet-induced obesity.

As one of insulin-sensitizing adipocytokines[27], adiponectin is the most abundant adipokine in the circulatory system and thought to be an important indicator of adipose function. It has been shown to stimulate glucose utilization and fatty-acid oxidation by the activation of AMP-kinase signaling pathway[28], and other related signaling pathways[29]. Moreover, adiponectin also has anti-inflammatory effects[30]. Our results demonstrated that telmisartan could increase excretion of adiponectin and improve systemic and local adipose dysfunction in obese rats.

We used 8-week-old rats, which had reached sexual maturity in our study, so most of the added weight induced by dietary intervention likely come from adipose tissues rather than muscle and skeleton. We found that visceral fat including perirenal fat and epididymis fat were increased in the HF group. After differences in body weight was adjusted, the increase in perirenal fat was found to be more than that in epididymis fat. Interestingly, telmisartan had a stronger effect in perirenal fat than epididymis fat. Our RT-PCR results showed that telmisartan increased the levels of *adiponectin* mRNA expression in visceral fat, especially perirenal fat.

Visceral fat accumulation is a major determinant of increased risks for cardiovascular and metabolic diseases associated with obesity and metabolic syndromes[31],[32]. Obesity also leads to ectopic fat storage within or around organs[33],[34]. In addition, visceral fat around organs may modify organ function by physical compression or through various active substances secreted by adipocytes. In a study involving 151 T2DM subjects, perirenal fat was found to be an independent predictor of kidney dysfunction[17]. In another study, it was found that perirenal fat might compress renal vessels and renal parenchyma, causing elevations in renal interstitial hydrostatic fluid, and reductions in both renal blood and tubular flow rates[35]. In our study, telmisartan treatment significantly decreased visceral fat, especially perirenal fat. Moreover, we found significant positive correlation between blood pressure, SBP, DBP and perirenal fat. These data strongly suggested that perirenal fat may directly contribute to high-fat diet-induced hypertension. Importantly, we found that telmisartan treatment decreased perirenal fat and increased the levels of *adiponectin* mRNA, which may improve perirenal dysfunction.

Epididymis fat is another type of visceral fat. Our results indicated that epididymis fat might not contribute to high-fat diet-induced hypertension. Additionally, we found that epididymis fat secreted less adiponectin compared with perirenal fat after telmisartan treatment.

A concurrence of disturbed glucose metabolism, abdominal fat distribution, dyslipidemia and hypertension is prevalent now. In our study, we found that telmisartan treatment could prevent the damaging changes in adipose tissues and blood pressure caused by high-fat diet, suggesting that telmisartan may be especially applicable in the treatment of hypertension induced by abnormal metabolism. Especially, effects of telmisartan treatment were comprehensive, and it is more convenient for clinical use in obese hypertension patients.

In summary, telmisartan prevented high-fat diet-induced hypertension and improved insulin resistance. Perirenal fat might be important in the pathogenesis of high-fat diet-induced hypertension, and the effects of anti-hypertension medication may be mediated in part by decreasing perirenal fat and ameliorating visceral fat dysfunction.

To confirm the effects of perirenal fat and other visceral depot, we should make a thorough investigation as many compounding factors may reduce the reliability of waist circumference measurement and body mass index in estimating the risk for obesity. The measurement of perirenal fat by ultrasound is accurate, simple and rapid, and might be widely used in estimating the risk for obese hypertension and other metabolic disorders.
